# Acute Toxicities of the Saxitoxin Congeners Gonyautoxin 5, Gonyautoxin 6, Decarbamoyl Gonyautoxin 2&3, Decarbamoyl Neosaxitoxin, C-1&2 and C-3&4 to Mice by Various Routes of Administration

**DOI:** 10.3390/toxins9020073

**Published:** 2017-02-21

**Authors:** Andrew I. Selwood, Craig Waugh, David T. Harwood, Lesley L. Rhodes, John Reeve, Jim Sim, Rex Munday

**Affiliations:** 1 Cawthron Institute, Private Bag 2, Nelson 7042, New Zealand; craig.waugh@cawthron.org.nz (C.W.); tim.harwood@cawthron.org.nz (D.T.H.); lesley.rhodes@cawthron.org.nz (L.L.R.); 2 Ministry for Primary Industries, P.O. Box 2526, Wellington 6140, New Zealand; john.reeve@mpi.govt.nz (J.R.); jim.sim@mpi.govt.nz (J.S.); 3 AgResearch Limited, Ruakura Research Centre, Private Bag 3123, Hamilton 3240, New Zealand; rex.munday@agresearch.co.nz

**Keywords:** paralytic shellfish toxins, gonyautoxins, decarbamoyl neosaxitoxin, decarbamoyl gonyautoxins, C1&2, C3&4, acute toxicity, toxicity equivalence factors, oral exposure

## Abstract

Paralytic shellfish poisoning results from consumption of seafood naturally contaminated by saxitoxin and its congeners, the paralytic shellfish toxins (PSTs). The levels of such toxins are regulated internationally, and maximum permitted concentrations in seafood have been established in many countries. A mouse bioassay is an approved method for estimating the levels of PSTs in seafood, but this is now being superseded in many countries by instrumental methods of analysis. Such analyses provide data on the levels of many PSTs in seafood, but for risk assessment, knowledge of the relative toxicities of the congeners is required. These are expressed as “Toxicity Equivalence Factors” (TEFs). At present, TEFs are largely based on relative specific activities following intraperitoneal injection in a mouse bioassay rather than on acute toxicity determinations. A more relevant parameter for comparison would be median lethal doses via oral administration, since this is the route through which humans are exposed to PSTs. In the present study, the median lethal doses of gonyautoxin 5, gonyautoxin 6, decarbamoyl neosaxitoxin and of equilibrium mixtures of decarbamoyl gonyautoxins 2&3, C1&2 and C3&4 by oral administration to mice have been determined and compared with toxicities via intraperitoneal injection. The results indicate that the TEFs of several of these substances require revision in order to more accurately reflect the risk these toxins present to human health.

## 1. Introduction

Paralytic shellfish poisoning (PSP) is a serious and sometimes fatal outcome of the consumption of seafood contaminated with saxitoxin and its congeners, which are produced by marine dinoflagellates of the genera *Alexandrium*, *Gymnodinium* and *Pyrodinium* and by several genera of freshwater cyanobacteria [1,2]. The geographic distribution of PSP-inducing organisms is increasing, and on a global scale, around 2000 cases of PSP are reported each year, with a mortality rate of 15% [3].

For many years, evaluation of the safety of seafood for human consumption has been based on a mouse bioassay (MBA), which involves intraperitoneal injection of an extract of the seafood in mice, with death as the endpoint. This assay has been approved as a reference method for paralytic shellfish toxins by the Association of Official Analytical Chemists [4]. Such an assay is, however, deemed by many to be ethically unacceptable and, further, its validity is questionable since it involves intraperitoneal injection rather than the oral route through which humans are exposed to the PSP toxins. The use of the MBA is now being phased out in several countries, and alternative chemical and functional assays for the paralytic shellfish toxins have been subjected to interlaboratory validations and approved by AOAC following review. These include two HPLC fluorescence methods [5,6], one using pre-column oxidation (AOAC 2005.06) and the other using post-column oxidation (AOAC 2011.02). Both of these methods allow quantitation of individual saxitoxin analogues present in a sample. A receptor binding assay has also been validated and approved (AOAC 2011.27) which determines a composite measure of sample toxicity based on the ability of sample extracts to compete with radiolabeled saxitoxin for binding to voltage-gated sodium channels [7]. 

As of 2010, more than 50 analogues of saxitoxin had been identified [8]. Instrumental methods for the quantitation of saxitoxin and many of its congeners in seafood are now available. Such methods permit the assessment of the concentration of the individual toxins in a seafood sample and this, together with knowledge of the relative toxicity of the various compounds, permits the overall toxicity of the sample to be determined, enabling assessment of the potential risk to human health.

The relative toxicities of saxitoxin congeners are expressed as “Toxicity Equivalence Factors” (TEFs), which define the toxicities of these substances as a ratio of that of saxitoxin itself. Again, an MBA has been used for the estimation of TEFs for saxitoxin congeners. An assay for saxitoxin itself was developed by Sommer and Meyer in the 1930s [9], based on the relationship between the dose of pure saxitoxin administered to mice by intraperitoneal injection and the time to death of the animals. The amount of saxitoxin in the sample injected, expressed as “Mouse Units”, was determined from the table of death-times established by these authors. Although validated only for saxitoxin itself, this MBA has more recently been applied to saxitoxin congeners, and TEFs for such congeners have been estimated from this data [10].

The validity of this approach is questionable. The assay depends upon intraperitoneal injection which negates the role the digestive system may play in either detoxifying some compounds, or in some cases, increasing their toxicological effect. Furthermore, the MBA is a bioassay, not a toxicological parameter, and it has been shown that TEFs derived from this method do not correlate with those derived from median lethal doses determined by approved toxicological methods [11]. The use of the MBA also assumes that the dose death-time relationships for saxitoxin congeners are the same as that for saxitoxin itself. This too has been shown to be untrue [11]. The inadequacy of the present TEFs for risk assessment was noted in the Scientific Opinion of the European Food Safety Authority Panel on Contaminants in the Food Chain, which indicated the need for establishing robust TEFs based on the relative oral toxicities of the saxitoxin congeners [10]. In a recent Expert Panel review of TEFs [12], it was agreed that the most relevant parameter for their determination was relative toxicity by oral administration and the Expert Panel recommended revisions to the presently used TEFs for certain saxitoxin congeners. Oral toxicity data are now available for neosaxitoxin, decarbamoyl saxitoxin, gonyautoxins 1&4 and gonyautoxins 2&3 [11]. As a continuation of these studies, we now report the acute toxicities of gonyautoxin 5 (GTX5), gonyautoxin 6 (GTX6), decarbamoyl gonyautoxin 2&3 (dcGTX2&3), decarbamoyl neosaxitoxin (dcNeoSTX), *N*-sulfocarbamoyl gonyautoxin 2&3 (C1&2) and *N*-sulfocarbamoyl gonyautoxin 1&4 (C3&4) by two methods of oral administration and a comparison of these data with the acute toxicities of these substances by intraperitoneal injection. The objective of this study is to add to the list of published TEFs for saxitoxin congeners based on oral administration in order to provide more robust TEF data applicable to the way in which humans are usually exposed to the major saxitoxin congeners found in seafood. 

## 2. Results

Details of the time to onset of symptoms, mortalities, death times and recovery times of mice dosed with the saxitoxin derivatives by all routes of administration are given as Supplementary Material (Table S1).

### 2.1. Acute Toxicity by Intraperitoneal Injection

The median lethal doses of the test substances by intraperitoneal injection are shown in Table 1. At lethal doses of the test compounds, the mice became lethargic within minutes after dosing, with rapid abdominal breathing. They subsequently became immobile. Their respiration became irregular and the rate of respiration declined. Respiration rates continued to decrease until breathing ceased completely. Exophthalmia and cyanosis were observed shortly before death, which occurred within 20 min of dosing with all congeners except for the relatively non-toxic C3,4. At sublethal doses, mice became lethargic, with abdominal breathing, and at doses close to the LD_50_, a decrease in respiration rate was also observed. The animals recovered over a period of 1–5 h, and their appearance and behavior remained normal throughout the subsequent 14-day observation period. No abnormalities were observed in any of the animals at necropsy.

### 2.2. Acute Toxicities by Oral Administration

The median lethal doses and the No Observable Adverse Effect Levels (NOAELs) of the test compounds by gavage are shown in Table 2. Those by feeding are given in Table 3.

The symptoms of intoxication via the oral route were the same as those recorded after intraperitoneal injection, although the time to onset of the changes was greater, with signs of intoxication appearing at up to an hour after dosing by gavage, and longer after administration by feeding. Death times were also extended. dcNeoSTX was unusual in that signs of intoxication were not observed at up to 3 h, and deaths were seen at up to 9 h after dosing. Time to recovery after sublethal doses of the toxins was also extended, and recover was incomplete at up to 9 h after dosing, particularly in mice dosed orally with GTX5, GTX6 and dcNeoSTX. 

### 2.3. Specific Activities of C1&2, C3&4 and dcNeoSTX by the MBA

The specific activities of C1&2, C3&4 and dcNeoSTX were 367, 69.5 and 43.0 MU/µmol, respectively. This compares to a value of 2090 MU/μmol for saxitoxin [11].

## 3. Discussion

As expected, the acute toxicities of the saxitoxin congeners by gavage were lower than those by intraperitoneal injection, most likely due to slower absorption via the oral route. Materials injected intraperitoneally are generally rapidly and extensively absorbed, leading to high tissue levels and toxicity. Slower absorption via oral administration may allow more time for detoxification and/or excretion of the test material before toxic levels are reached. It should be noted, however, that there were wide variations in the ratios between the toxicities by the two routes of administration. This difference was most pronounced with dcNeoSTX, which showed one of the lowest toxicities by injection, but the highest by gavage and by feeding. 

It has been argued that administration by feeding, rather than by gavage, is the most relevant route for toxicity determinations in rodents, since the semi-solid content of the stomach of these animals does not permit mixing of the material given by gavage, which may flow around the stomach contents and rapidly enter the duodenum. When given by feeding, however, the test material becomes mixed with the stomach contents of rodents in the same way that substances are distributed throughout the liquid contents of the human stomach, leading to relatively slow release into the absorptive areas of the gastrointestinal tract [13]. This is consistent with the observation that the absolute values of the acute toxicities of the saxitoxin derivatives were lower by feeding than by gavage. The ratio between the toxicity by feeding and that by gavage ranged from 2.1 to 2.6 for C1&2, GTX5 and dcNeoSTX, which is consistent with results with other saxitoxin congeners [11]. The ratios for dcGTX2&3 and GTX6 were higher, however (4.1 and >6, respectively). The reason for this disparity is not presently known. Possibilities include the conversion of these compounds into less toxic substances during the relatively long residence time in the stomach of the animals or the inhibition of stomach contraction or of the opening of the pyloric sphincter, leading to slower release into the duodenum. 

For accurate risk assessment, it is essential that relevant and accurate TEFs for saxitoxin and its congeners are available. At present, the relative risk to human health of saxitoxin derivatives is largely based on TEFs calculated from the specific activities of these substances determined in the MBA. As shown previously [11], the relative acute toxicities of a number of saxitoxin congeners by intraperitoneal injection do not correlate with their relative specific activities in the MBA. This is consistent with the observation that the death time-dose curves for the saxitoxin derivatives are not the same as that for saxitoxin itself [11].

In the present study, the acute toxicities of GTX5, GTX6, dcGTX2&3, dcNeoSTX, C1&2 and C-3&4 were determined. MBA data are available for GTX5, GTX6 and dcGTX2&3 [10]. No MBA data on epimeric mixtures of C1&2 or C3&4 are available. Also, the MBA figure given by European Food Safety Authority (EFSA) for dcNeoSTX [10] is regarded as incorrect. The figure given is that from Sullivan et al. [14], though these authors did not determine the specific activity of dcNeoSTX, but assumed that it was the same as that of decarbamoyl saxitoxin. In order to facilitate comparison, we determined the specific activities of the C-toxin equilibrium mixtures and that of dcNeoSTX. It should be noted that the equilibrium mixtures of the epimers of dcGTX2&3, C1&2 and C3&4 were evaluated in these studies, rather than the individual epimers, since the latter substances are never found in isolation in seafood, but invariably as equilibrium mixtures.

A comparison of the TEFs derived from the MBA, acute toxicity by intraperitoneal injection and by oral administration of the above toxins is shown in Table 4. Again, there was no correlation between the TEFs derived by the MBA and those from acute toxicity by intraperitoneal injection. The TEFs based on the MBA were similar to those based on oral toxicity for GTX5 and C3&4, but were higher for GTX6, dcGTX2&3 and C1&2 and lower for dcNeoSTX. The TEFs based on toxicity by feeding were ~40% lower than those proposed by EFSA for GTX5 and dcNeoSTX, and more than five times lower for GTX6.

The results of the present study suggest that the currently used TEFs for some of the above compounds should be revised based on the available oral toxicity data, and this has been recommended in a recent Expert Panel review [12]. In this way, appropriate regulatory limits can be set that are not so high as to endanger human health and not so low that they cause unnecessary loss to the seafood industry through destruction of product or closure of harvesting areas.

## 4. Materials and Methods

### 4.1. Purification and Analysis of Toxins

**Figure 1:**
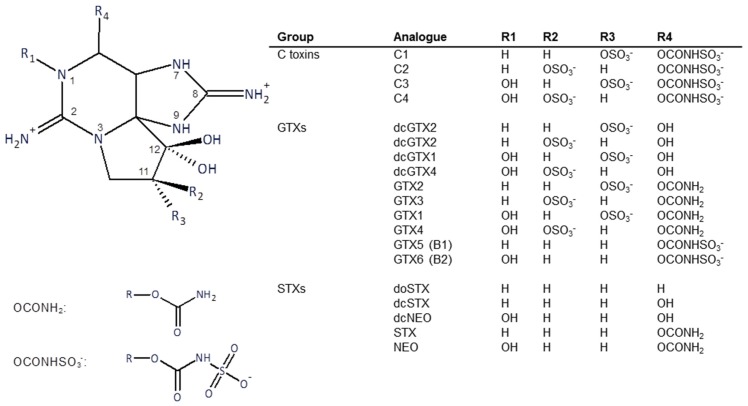
Structure of the major paralytic shellfish toxins.

Structures of the PSTs are shown in Figure 1. The toxins used in this study were purified from *Alexandrium catenella* cells collected from a bloom event that occurred in Opua Bay, Marlborough Sounds, New Zealand, in 2013. The toxins were extracted and purified using preparative column chromatography and chemically converted to other analogues as necessary, using techniques previously described [15,16]. Briefly, for toxin isolation, bulk cultures of *A. catenella* were extracted with hot dilute acetic acid. Cell debris was removed by centrifugation and filtration. The toxins were recovered using activated carbon column chromatography. Further purification used gel filtration and ion-exchange chromatography. 

**Figure 2:**
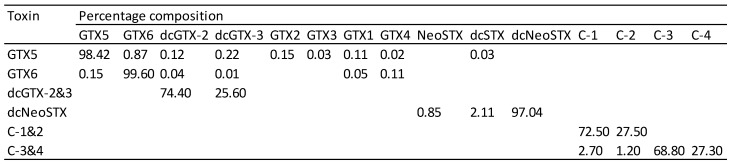
Percentage molar concentration of PSTs in test materials

The purified toxins were dissolved in 10 mM acetic acid to give concentrated stock solutions. Dilutions of these solutions were accurately prepared volumetrically, with purity and concentration determined using liquid chromatography with fluorescence detection [6] and liquid chromatography with mass spectrometric detection [17]. National Research Council of Canada (NRC) certified reference materials (CRMs) were used as calibrants for all of the toxins generated except for C3&4 and GTX6, for which no CRMs were available. Instead, C3&4 was quantified by measuring the concentration of GTX1&4 formed by acid hydrolysis [18,19] using the conversion of C1&2 to GTX2&3 as a control. The concentration assigned from this approach was in good agreement with direct measurement using non-certified C3&4 reference materials from NRC and the Japanese National Research Institute of Fisheries Science. GTX6 was quantified directly using a non-certified reference material from NRC and confirmed by quantifying neoSTX generated by acid hydrolysis. C1&2, C3&4, and dcGTX2&3, exist as pairs of epimers. These mixtures were equilibrated prior to toxicological analysis to give a ratio of approximately 3:1 (Figure 2). This represents the same ratio that is found in shellfish contaminated with these toxins.

### 4.2. Animals

Female Swiss albino mice, bred at AgResearch, Ruakura, New Zealand, were employed in all experiments. The initial body weights of the mice were between 18 and 22 g. They were housed in solid-bottomed cages containing bedding of softwood shavings. The animals were allowed unrestricted access to food (Rat and Mouse Cubes, Speciality Feeds Ltd., Glen Forrest, Western Australia) and tap water throughout the experimental period. All experiments were approved by the Ruakura Animal Ethics Committee, Approval Number 12327 3/10/2013 and 13371, 2/10/2014.

### 4.3. Determination of Median Lethal Doses

Acute toxicities were determined using the up-and-down procedure according to the principles of OECD Guideline 425 [20]. Mice were weighed immediately before dosing, and the test substances were administered on a µmol/kg body weight basis. Aliquots of the test materials were diluted in 3 mM HCl. For intraperitoneal injection, the volume administered was 1 mL, while for gavage the volume was 200 µL. For determination of toxicity by feeding, mice were trained to eat small amounts of cream cheese, as described previously [11]. For dosing, toxins, in solution in 3 mM HCl, were mixed with ~150 mg of cheese and immediately fed to the mice, who readily ate the food within 45 s. In order to avoid diurnal variations in response, dosing by all routes of administration was conducted between 8.00 and 9.30 a.m. The mice were monitored intensively during the day of dosing. Those dying during the course of the experiments were necropsied, while survivors were weighed and examined each day for 14 days, after which time they were killed by carbon dioxide inhalation and necropsied. 

### 4.4. Determination of the No Observable Adverse Effect Levels (NOAELs)

Mice were dosed by gavage or by feeding with the test materials at doses below the LD_50_. A logarithmic dose-progression was employed, using the protocol of OECD Guideline 425, but with “toxic effect” rather than death as the parameter. Exploratory behavior was assessed by transferring the mice to a new cage and observing their movements. Abdominal breathing and lethargy were assessed visually.

### 4.5. Determination of the Specific Activities of C-1*&*2, C-3*&*4 and dcNeoSTX by the MBA

Aliquots of the test materials, diluted to 1 mL with 3 mM HCl, were injected intraperitoneally in mice according to the protocol of AOAC Official Test Method 959.08 [4]. Median death times were calculated, and MU/mL determined from Table 959.08A in the AOAC method. Specific activities were calculated as MU/µmol.

## Figures and Tables

**Table 1:** Acute toxicities of the test substances by intraperitoneal injection.

Compound	LD_50_ (µmol/kg) *
Saxitoxin **	0.028 (0.025–0.031)
GTX5	0.125 (0.065–0.155)
GTX6	0.227 (0.173–0.277)
dcGTX-2&3	0.040 (0.032–0.050)
dcNeoSTX	0.478 (0.439–0.493)
C1&2	0.400 (0.327–0.663)
C3&4	0.480 (0.472–0.500)

* Figures in brackets indicate 95% confidence limits; ** Data from Reference [11].

**Table 2:** Acute toxicities and NOAELs of the test substances by gavage.

Compound	LD_50_ (µmol/kg) *	NOAEL (µmol/kg) *
Saxitoxin **	1.19 (1.02–1.30)	0.544 (0.500–0.560)
GTX5	18.9 (14.1– 21.7)	5.12 (4.80–6.00)
GTX6	31.1 (29.5–36.5)	7.90 (7.42–9.31)
dcGTX2&3	7.13 (6.00–7.60)	2.53 (2.38–3.00)
dcNeoSTX	5.50 (4.13–6.34)	2.13 (1.96–2.20)
C1&2	35.0 (30.6–46.7)	15.0 (10.5–19.9)
C3&4	42.7 (40.0–50.0)	25.5 (23.8–30.0)

* Figures in brackets indicate 95% confidence limits; ** Data from Reference [11].

**Table 3:** Acute toxicities and NOAELs by feeding.

Compound	LD_50_ (µmol/kg) *	NOAEL (µmol/kg) *
Saxitoxin **	3.20 (2.20–4.27)	ND
GTX5	50.0 (37.5–72.9)	17.1(16.0–20.1)
GTX6	>188	ND
dcGTX2&3	29.6 (25.0–32.0)	10.0 (7.01–13.4)
dcNeoSTX	14.3 (10.8–15.9)	4.36 (4.00–4.49)
C1&2	74.0 (69.0–87.0)	17.4 (8.93–21.6)
C3&4	ND	ND

* Figures in brackets indicate 95% confidence limits; ** Data from Reference [11]; ND, Not determined.

**Table 4:** Comparison of TEFs derived from the MBA, i.p. injection and oral administration.

Compound	TEF Proposed by EFSA (EFSA 2009)	TEF Based on MBA	TEF Based on LD_50_ by i.p. Injection	TEF Based on LD_50_ by Gavage (This Study)	TEF Based on LD_50_ by Feeding (This Study)
Saxitoxin	1.0	1.0	1.0	1.0	1.0
GTX5	0.1	0.06 [10]	0.22	0.063	0.064
GTX6	0.1	0.08 [10]	0.12	0.038	<0.017
dcGTX2&3	-	0.19 [10]	0.70	0.17	0.11
dcNeoSTX	0.4	0.021 (This study)	0.058	0.22	0.22
C1&2	-	0.18 (This study)	0.070	0.034	0.043
C3&4	-	0.033 (This study)	0.058	0.028	ND

## Supplementary Materials

The following are available online at www.mdpi.com/2072-6651/9/2/73/s1, Table S1: Time to onset of symptoms, mortalities, death times and recovery times of mice dosed with the saxitoxin derivatives.
