# Supplementary material for: Acute Toxicities of the Saxitoxin Congeners Gonyautoxin 5, Gonyautoxin 6, Decarbamoyl Gonyautoxin 2&3, Decarbamoyl Neosaxitoxin, C-1&2 and C-3&4 to Mice by Various Routes of Administration
Source: Toxins (Basel). 2017 Feb 21;9(2):73. doi: 10.3390/toxins9020073 (PMC5331452; doi:10.3390/toxins9020073)
Supplement: Supplementary file 1 [file toxins-09-00073-s001.pdf]

Supplementary Materials: Acute Toxicities of the Saxitoxin Congeners Gonyautoxin 5, Gonyautoxin 6, Decarbamoyl Gonyautoxin 2&3, Decarbamoyl Neosaxitoxin, C-1&2 and C-3&4 to Mice by Various Routes of Administration

Andrew I. Selwood, Craig Waugh[a], David T. Harwood, Lesley L. Rhodes, John Reeve, Jim Sim and Rex Munday

Table S1. Time to onset of symptoms, mortalities, death times and recovery times of mice dosed with the saxitoxin derivatives.

GTX5

Acute toxicity by intraperitoneal injection

Dose (µmol/kg)	Total number of mice	Time to onset of symptoms (minutes)	Number of dead mice	Time of death (minutes)	Number of surviving mice	Time to recovery (minutes)
0.100	1	6	0	-	1	48
0.125	2	2, 3	1	7	1	78
0.158	2	1, 2	2	7, 7	0	-
0.199	1	3	1	6	0	-

Acute toxicity by gavage

Dose (µmol/kg)	Total number of mice	Time to onset of symptoms (minutes)	Number of dead mice	Time of death (minutes)	Number of surviving mice	Time to recovery (minutes)
12.6	1	-	0	-	1	90
15.0	2	8, 10	0	-	2	125, 175
18.9	3	13, 15, 20	2	161, 255	1	247
23.8	2	5, 8	2	125, 147	0	-

Acute toxicity by feeding

Dose (µmol/kg)	Total number of mice	Time to onset of symptoms (minutes)	Number of dead mice	Time of death (minutes)	Number of surviving mice	Time to recovery (minutes)
40.0	1	29	0	-	1	327
50.0	3	23, 28, 30	1	152	2	142, 305
64.0	2	20, 23	2	77, 74	-	-

C1&2

Acute toxicity by intraperitoneal injection

Dose (µmol/kg)	Total number of mice	Time to onset of symptoms (minutes)	Number of dead mice	Time of death (minutes)	Number of surviving mice	Time to recovery (minutes)
0.250	1	2	0	-	1	46
0.320	2	3, 4	0	-	2	103, 105
0.400	2	2, 3	1	11	1	110
0.449	1	3	1	19	0	-

Acute toxicity by gavage

Dose (µmol/kg)	Total number of mice	Time to onset of symptoms (minutes)	Number of dead mice	Time of death (minutes)	Number of surviving mice	Time to recovery (minutes)
28.0	2	26, 31	0	-	2	118
35.0	3	16, 19	1	32	2	182
44.0	2	7, 17	2	85, 98	0	-

Acute toxicity by feeding

Dose (µmol/kg)	Total number of mice	Time to onset of symptoms (minutes)	Number of dead mice	Time of death (minutes)	Number of surviving mice	Time to recovery (minutes)
55.0	1	42	0	-	1	142
69.0	2	30, 45	0	-	2	255, 617
87.0	3	21, 27	3	173, 72, 110	-	-

GTX6

Acute toxicity by intraperitoneal injection

Dose (µmol/kg)	Total number of mice	Time to onset of symptoms (minutes)	Number of dead mice	Time of death (minutes)	Number of surviving mice	Time to recovery (minutes)
0.119	1	10	0	-	1	185
0.150	2	2, 9	0	-	2	70, 78
0.189	3	1, 2	2	5, 10	1	181
0.238	1	2	1	4	-	-

Acute toxicity by gavage

Dose (µmol/kg)	Total number of mice	Time to onset of symptoms (minutes)	Number of dead mice	Time of death (minutes)	Number of surviving mice	Time to recovery (minutes)
25.0	2	17, 30	0	-	2	>535, > 382
31.0	3	15, 22, 25	3	52, 80, 80	0	-
40.0	1	21	1	90	0	-

Acute toxicity by feeding

Dose (μmol/kg)	Total number of mice	Time to onset of symptoms (minutes)	Number of dead mice	Time of death (minutes)	Number of surviving mice	Time to recovery (minutes)
94.0	1	85	-	-	1	303
119	1	80	-	-	1	400
151	1	46	-	-	1	392
188	1	53	-	-	1	347

dcGTX2&3

Acute toxicity by intraperitoneal injection

Dose (μmol/kg)	Total number of mice	Time to onset of symptoms (minutes)	Number of dead mice	Time of death (minutes)	Number of surviving mice	Time to recovery (minutes)
0.032	1	12	0	-	1	123
0.040	3	4, 9, 11,	1	9	2	205, 312
0.050	2	2, 3	2	7, 11	0	-
0.063	1	4	1	6	0	-
0.079	1	2	1	6	0	-

Acute toxicity by gavage

Dose (μmol/kg)	Total number of mice	Time to onset of symptoms (minutes)	Number of dead mice	Time of death (minutes)	Number of surviving mice	Time to recovery (minutes)
4.80	1	62	0	-	1	202
6.00	3	10, 15, 53	0	-	3	120, 105, 218
7.60	2	9, 10	2	15, 52	0	-

Acute toxicity by feeding

Dose (μmol/kg)	Total number of mice	Time to onset of symptoms (minutes)	Number of dead mice	Time of death (minutes)	Number of surviving mice	Time to recovery (minutes)
15.9	1	34	0	-	1	85
25.0	3	34, 55, 113	0	-	3	200, 270, 138
32.0	2	31, 72	2	147, 82	0	-

dcNeoSTX

Acute toxicity by intraperitoneal injection

Dose (µmol/kg)	Total number of mice	Time to onset of symptoms (minutes)	Number of dead mice	Time of death (minutes)	Number of surviving mice	Time to recovery (minutes)
0.277	1	8	0	-	1	449
0.311	1	6	0	-	1	296
0.349	1	10	0	-	1	377
0.391	1	7	0	-	1	342
0.439	3	4, 8, 16	0	-	3	309, 470, 522
0.493	2	2, 3	2	50, 180	0	-

Acute toxicity by gavage

Dose (µmol/kg)	Total number of mice	Time to onset of symptoms (minutes)	Number of dead mice	Time of death (minutes)	Number of surviving mice	Time to recovery (minutes)
3.50	1	105	0	-	1	152
4.40	2	95, 181	0	-	2	305, 371
5.50	3	53, 87, 132	2	435, 380	1	531
7.00	2	100, 190	2	337, 290	0	-

Acute toxicity by feeding

Dose (µmol/kg)	Total number of mice	Time to onset of symptoms (minutes)	Number of dead mice	Time of death (minutes)	Number of surviving mice	Time to recovery (minutes)
12.8	1	68	0	-	1	382
14.3	2	85, 110	1	354	1	>443
16.1	2	53, 90	2	425, 518	0	-
18.0	1	40	1	250	0	-
20.2	1	133	1	219	0	-

C3&4

Acute toxicity by intraperitoneal injection

Dose (µmol/kg)	Total number of mice	Time to onset of symptoms (minutes)	Number of dead mice	Time of death (minutes)	Number of surviving mice	Time to recovery (minutes)
0.446	1	8	0	-	1	141
0.472	2	8, 9	0	-	2	108, 117
0.500	3	3, 3, 5,	3	20, 31, 43	0	-

Acute toxicity by gavage

Dose (µmol/kg)	Total number of mice	Time to onset of symptoms (minutes)	Number of dead mice	Time of death (minutes)	Number of surviving mice	Time to recovery (minutes)
40	2	26, 33,	0	-	-	240, 80
50	3	10, 12, 17	3	46, 52, 44	-	-
